# A Cost–Benefit Analysis of Preparing National Veterinary Services for Transboundary Animal Disease Emergencies

**DOI:** 10.1155/2023/1765243

**Published:** 2023-11-04

**Authors:** William Gilbert, David Adamson, Daniel Donachie, Keith Hamilton, Jonathan Rushton

**Affiliations:** ^1^Institute of Infection, Veterinary and Ecological Sciences, University of Liverpool, Liverpool, UK; ^2^School of Economics and Public Policy, University of Adelaide, Adelaide, Australia; ^3^World Organisation for Animal Health, Paris, France

## Abstract

The natural, accidental, or deliberate release of pathogens into livestock populations carries with it a range of consequences for society, from zoonotic disease outbreaks, to changes in food security and economic welfare. An important contribution to mitigating the risk of disease outbreaks comes from having well-prepared emergency response plans and agencies with the capacity to put those plans into operation. In the case of animal disease, national Veterinary Services (VS) take a central role. Unknown and uncertain events, such as if, when and where the next disease outbreak will occur makes economic decision-making a challenge. While the costs of preparing for emergencies can be quantified in a conventional manner, the scope, scale, and likelihood of benefits actually accruing are all subject to uncertainty. This study attempts to examine the costs and benefits of preparing national VS for animal disease emergencies, including natural, accidental, or deliberate release of pathogens. Data collected as part of the World Organisation for Animal Health's Performance of VS program for countries in East and West Africa and South East Asia were used for estimating investment costs. A state-contingent approach is used to constrain the uncertainty space in terms of disease impact. The probability of a disease event occurring and the probability of that event being contained by emergency preparation are used to describe a frontier at which investment breaks-even in a variety of scenarios. An increased probability of breaking-even on investment was found with high livestock numbers per capita and increasing intensification in livestock production systems. The method and findings provide a means to understand the benefits of preparing for uncertain events and are aimed to further the dialogue around policy development for livestock disease emergencies in lower-income countries.

## 1. Introduction

The impact of transboundary diseases on human health, nutrition, and prosperity has been brought sharply into relief with the coming of the COVID-19 pandemic in 2020. In a globalized, interconnected world, local failure to contain contagious disease can have far-reaching and catastrophic consequences.

The allocation of resources to prepare for disease-related emergencies is now a question of international concern. Considering the impacts of animal diseases on food supply, economies, and livelihoods, and with three quarters of emerging infectious diseases of humans being of animal origin [1], this concern extends to disease control in animal populations.

Animals, and in particular livestock, play important roles in society. In the developed world, highly productive livestock populations exist which are very often free from the major transmissible diseases. Disease outbreaks in these naïve populations can be socially and economically catastrophic, resulting from both the direct impacts of disease and the costs of reestablishing disease freedom [2]. Elsewhere in the world, animals are often integral to food security, not only as a source of protein but also as part of mixed crop-livestock systems [3].

Maintaining the health of these livestock populations is a constant challenge. The natural, accidental, or deliberate introduction of new pathogens to livestock populations has the potential to cause diverse economic, social, environmental, and cultural shocks in both the short and long term. Shifts in land-use across the globe have brought humans and livestock into contact with wildlife with a consequential risk of pathogen emergence [4]; changes in climate are driving disease vectors to alter their range [5], and movements of animals through trade risk introducing disease to new regions [6, 7]. Against this background, the purposeful release of a pathogen into livestock populations as a criminal act, an act of warfare, or one of terrorism has been identified as a risk to which the livestock sector is vulnerable for several reasons [8]. While the socioeconomic severity of livestock disease epidemics has already been discussed, the agents required to produce such outbreaks are relatively easy to acquire and amplify, safe to handle if not zoonotic, and introduction does not require highly sophisticated delivery mechanisms [9].

Significant levels of resource are devoted by national governments to quarantine and border control, risk analysis, and other activities to mitigate against the risk of disease introduction. Contingency plans are also prepared, acknowledging that risk mitigation can fail and disease outbreaks will occur [10]. Both developed and developing economies, therefore, have an interest in preparing for animal disease emergencies to protect themselves from the socioeconomic consequences of disease outbreaks and to ensure the food and nutritional security of their populations. It then remains to decide at what level resources should be allocated to this task. Mounting an effective response to disease outbreaks often requires the collaboration, funding, and coordination of multiple agencies and organizations across the public and private sectors. Recent investigations have revealed the vulnerability of emergency preparation to lack of funding [11].

Under the resource constraints, endemic pathogens are often prioritized since their impact is better understood, while extraordinary events are by their nature hard to anticipate and conceive of their impact. This question, on the impact of extraordinary events, is one that can be studied through an economic lens by measuring the cost of emergency preparation against the costs of a putative animal health emergency.

Veterinary Services (VS) are the governmental and nongovernmental organizations that implement animal health and welfare measures and are critical to preparing and responding to livestock diseases [12, 13]. Within VS there are multiple areas of competency, some of which are directly relevant to emergency response, some of which are transferable to emergency response depending on priorities, and others which bear little relevance to emergency preparation. It is hypothesized that a VS that is well-prepared and provisioned has the capacity to respond more efficiently and effectively to an emergency event than one that is poorly prepared and provisioned. This is represented by a greater probability of containing a disease outbreak, thereby reducing the total size of the outbreak due to resources already being allocated to deal with such events [13].

Quantifying what this means is a complex task when the range of pathogens capable of causing an emergency event is broad and includes novel or previously unknown agents, the means of introduction include natural and manmade pathways, and the populations at risk include multiple species and the timeframe over which events may occur is unknown [14]. On the cost side, preparing for emergency events involves building and maintaining infrastructure, human capital and crosscutting competencies in multiple institutions and agencies, and distributing costs across multiple budgets [15]. Furthermore, these kinds of investments are likely to yield benefits in other nonemergency activities that are difficult to track and quantify. In addition to uncertainty over the costs and benefits, the scope of emergency events and response means the data required to perform economic analysis may be hard to locate, acquire, and analyze.

The result is an environment of significant uncertainty in which evidence is needed to support policymaking. Adamson et al. [16] present this space as one in which, when emergencies are distributed according to impact, low-impact events occur at the highest frequency, while the highest-impact events occur at the lowest frequency. Due to the effect these rare, high-impact events can have on peoples' lives, it is they that are of principal interest to policy makers; the authors therefore argue for placing limits on the uncertainty space by using a state-contingent approach [17]. This method breaks a continuous set of uncertain outcomes into discrete states and then focusing analysis on the probability of forcing a transition between impact states. Describing the dynamic between investment costs, likelihood of event and magnitude of impact creates an analytical framework that policy makers can consider against the social, political, and economic realities of their own national context.

With this background information in mind, the following research questions arise:

1. Given an increase in the level of investment in emergency preparation, what probability of reduction in outbreak size is required to break-even against a greater cost of investment?
2. How many outbreaks must occur over the time period of an investment to justify the increased spending?

The paper presents an application of cost–benefit analysis to investing in emergency preparation using a state-contingent approach to uncertainty. This is achieved by the following objectives: first, the costs of investing in emergency preparation are quantified from the perspective of VS in four geographical regions of focus. Second, the potential impacts of animal health emergencies are placed into distributions and classed into discrete states of nature. Finally, the costs and benefit estimates are combined in a cost–benefit analysis over a 10-year period incorporating the likelihood of transition between each state of nature. The analysis values the rate at which high-impact outbreaks are converted into limited outbreaks by a well-prepared emergency response in order to justify increased levels of investment in the EP of VS. To facilitate the interpretation the results are presented as a probability frontier.

## 2. Materials and Methods

In consultation with stakeholders, four global regions of interest were defined for the study: East Africa, West Africa, the Middle East and South East Asia and to include only mainland countries since islands are exposed to a different level of disease risk by virtue of their geography. Following initial data scoping exercises, the Middle East region presented significant data gaps on prior and future emergency preparation costs and was excluded. The results presented are restricted to the East and West African and South East Asian regions. For reasons of confidentiality, specific country results are anonymized, but overview characteristics in terms of GDP, livestock density, and VS funding are given in Table 1.

For each nation, the cost of preparing VS for emergency response and the economic loss caused by possible disease outbreak scenarios were estimated. This was done by defining three budgetary states (baseline, full investment, and partial investment) and three disease states (no disease, limited outbreak, and major outbreak) for each country. The size of each outbreak state was derived from actual outbreak data for three major livestock diseases, and their impact on commercial livestock production of pigs, poultry, and cattle in target countries estimated by economic model.

The detail of these methods is described in the following section.

### 2.1. Costing Emergency Preparation

The WOAH Performance of Veterinary Services (PVS) Pathway is WOAH's flagship capacity-building program for the sustainable improvement of the VS in WOAH members. It helps members to identify and understand their strengths and weaknesses of their compliance with WOAH standards. WOAH members can voluntary request participation in the program which includes the PVS Evaluation and PVS Evaluation Follow-up missions that use the PVS Tool [18], which can then be followed by PVS Gap Analysis to support members to develop goals, objectives, and a strategy for improvement accompanied by an investment plan. The PVS Tool divides the VS activities into 45 critical competencies (CCs) and assesses the performance of each one. The PVS Evaluation divides CCs into four domains: resourcing, technical authority and capability, stakeholder engagement, and access to markets. Within each domain, a number of activities are detailed which are then rated on a 1–5 scale. CCs relating to emergency preparedness (EP) were identified by consulting the FAO Good Emergency Management Protocol [15]. Country-level current and target scores for EP CCs were recorded for each country. Since its inception, the PVS framework has been through several revisions and therefore some equivalencies between CCs were required to standardize the data over time. The equivalencies between PVS versions are provided in Supplementary Table S1 accompanying this paper.

A PVS Gap Analysis supplements the PVS Evaluation with an action and budget plan aimed at addressing the needs identified in the PVS Evaluation over years. PVS has thereby created a dataset that may be unique in allowing costing of VS by activity across multiple countries, within a unifying framework. PVS Gap data gathered since 2007 was extracted from WOAH's database for the countries available within the regions of interest.

Budget data from PVS Gap are divided into two brackets: annual and extraordinary expenditure. Extraordinary expenditure consists of the capital, training, and consultation costs required to initiate improvements in selected CCs, while annual budgets are equivalent to maintenance budgets for assets, salaries, and consumables. For each country, reports provided full VS budgets across all competencies for both the pre-Gap status quo and the post-Gap investment plan.

All Gap budget data were transcribed into Microsoft Excel (Microsoft Corporation). The durability of assets, reporting currencies, exchange rates, and structure of CCs varied between individual reports, so the data required cleaning, reduction, and standardization to be aggregated.

Detailed annual and capital costs were available by CCs within the post-Gap investment plan. These were recorded. All figures were then adjusted for inflation according to World Bank Consumer Price Index data [19], and converted to purchasing power parity dollars (PPP$ 2017).

To standardize the lifetime of assets across the data, fixed capital items were placed into brackets of 5, 8, 10, 15, or 25 years according to expected useful lifetime, allowing straight-line depreciation to be applied to their values. The following budgetary variables were recorded for each country:

(1) Pre-Gap:
  - Full VS annual budget.
(2) Post-Gap:
  - Full VS annual budget recommendation.
  - Full VS (extraordinary) capital budget recommendation.
(3) Post-Gap priority CCs for EP:
  - EP-priority CC annual budget.
  - EP-priority CC capital budget.

The data structure provided the cost structure for the cost–benefit analysis scenarios: (1) a preinvestment baseline cost for VS, (2) a full investment scenario, where the entire VS budget follows the Post-Gap plan, and finally, (3) a targeted investment plan aimed at EP-priority CCs only.

Following the exclusion of countries with incomplete data, 18 countries in the regions of interest were retained. To represent uncertainty in the final budget estimates after adjustment for inflation and conversion to PPP$ the following ranges were applied to generate a conservative cost distribution:

1. Pre-Gap budget  ^*∗*^ 0.5 to Pre-Gap budget  ^*∗*^ 1 on the baseline scenario, representing a 50% underspend relative to the data.
2. Post-Gap budget  ^*∗*^ 1 to Post-Gap budget  ^*∗*^ 1.5 on the investment scenario, representing a 50% overspend relative to the estimated investment amount.

### 2.2. Quantifying the Benefits of Emergency Preparation

Estimating the benefits of disease control was based on the following process. First, describing the populations of animals present in each country of interest. Second, estimating the output of those populations over time and the value of animals present. Finally, estimating the effect of disease outbreaks on output and population values for different outbreak sizes.

#### 2.2.1. Description of Populations and Production

Data from FAOSTAT were used to define the size of livestock populations in each of the countries of interest in the year 2016 [20]. FAOSTAT also provided further data on quantities for domestic production for each of these species and products, slaughter statistics, producing animals for eggs and milk as a proportion of the total population, and per-animal yields. Trade data by country were extracted from the UN Comtrade database for bovines, swine, and poultry (live animals) and for each species meat, eggs, and milk products [21].

Gilbert et al. [22] showed that the proportion of livestock being raised in intensive systems can be predicted with a good degree of accuracy from per capita GDP levels, and this principle was applied to create a compartmental model of the population. Using World Bank GDP estimates for the year 2016 and a linear approximation of the model described by Gilbert et al. [22] produced transition from 5% to 95% of the total population into intensive compartments as GDP per capita increased from $1,000 to $30,000 PPP for pigs and from $1,000 to $10,000 PPP for chicken.

Within each country, simplified models of livestock production with populations assumed static over time were constructed in Microsoft Excel to allow calculations of disease impact to be performed.

**Figure 1:**
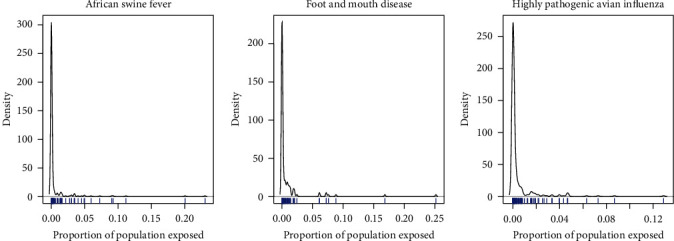
Proportion of the population exposed to African swine fever, foot and mouth disease, and highly pathogenic avian influenza for each outbreak reported to WOAH WAHIS 2015–2019. The frequency of observation density (*y*-axis) has a large positive skew, with larger outbreaks becoming increasingly infrequent as proportion of total population exposed increases. A rug plot (blue) running along the *x*-axis illustrates all the observations by their *x* coordinate, highlighting the location of extreme values.

For the poultry sector, the total population of chickens from FAOSTAT was separated into intensive and other systems and divided into production types according to the framework, as illustrated in Figure 1. The ratios in the layer and breeder sector were calculated from FAOSTAT data, and it was then assumed 10% of those birds would be breeders [23, 24]. Productivity per bird was estimated at 0.03 kg of eggs per bird day in the layer sector, 0.226 pullets per bird day in the breeder sector, and 0.014 broilers per placement day in the broiler sector [25–30]. In the cattle sector, population data were extracted from FAOSTAT and subject to no further treatment to model the different production systems. In the pig sector, population, slaughter, and live animal trade data were used to estimate pigs moving in and out of the system on an annual basis, from which the number of live pigs produced domestically was calculated. This number was then divided by an assumed ratio of sows (15%) and boars (1%) in the population, with the remainder being growers [31–36]. Growing pigs were valued based on average slaughter weight (FAOSTAT), with an assumed dressing percentage of 75%. The proportion of pigs living in commercial systems was estimated using the method of Gilbert et al. [22].

In order to reflect uncertainty when extrapolating literature and FAOSTAT data from local production systems across geographical regions and time, variation was included in the model to perform sensitivity analysis to changes in parameters This sensitivity analysis included the uncertainty over sector output prices (meat, milk, and eggs) and stock prices (replacement animals). Given the wide range of prices observed, a sensitivity of ±33% was placed on price data.

#### 2.2.2. Estimating Outbreak Size

To define a set of possible emergency-causing pathogens, the legal frameworks set up by various governments were examined as to which pathogenic organisms are subject to movement restriction and other controls [34–36]. Given the broad nature of the pathogen threats and risk pathways involved in the creation of animal health emergencies, three case study diseases were selected from this aggregated list to illustrate the potential benefits of effective emergency response, encompassing the major food-producing species:

- Foot and mouth disease (cattle).
- African swine fever (pigs).
- Avian influenza in intensive chicken systems.

These three viral diseases were chosen as they are amongst the most significant economic threats with respect to their ability to cause livestock morbidity and mortality and affect domestic and international trade of livestock products [2]. Additionally, HPAI has zoonotic and pandemic potential and poses a risk to human health and public health systems [37, 38].

For each of these diseases, a distribution of outbreak sizes was constructed from real outbreaks reported to the WOAH WAHIS database for the years 2015–2019 [39]. Outbreak sizes were recorded as number of animals exposed, and total species populations [20] were used as a denominator to estimate proportion of population exposed for each outbreak. Outbreak size followed an extreme-value distribution [40], as shown in Figure 1.

The following three outbreak states were defined from this distribution:

- no disease event,
- limited disease event, and
- major disease event.

A limited outbreak was set at the median point of the empirical distribution, while a major outbreak was classified as a rare event at the 99th percentile for proportion of population exposed and case rate within population at risk. Mortality rate and slaughter rate amongst cases were taken as the median of the empirical distribution for these variables (Table 2).

#### 2.2.3. Impact of Outbreak Scenarios

**Figure 2:**
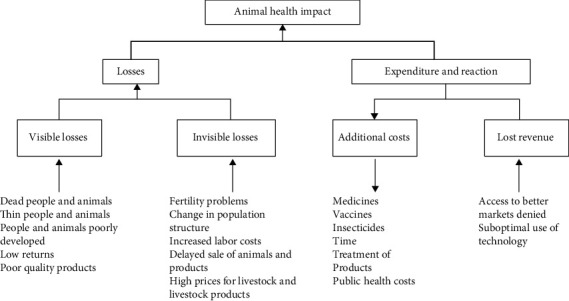
Framework for estimating the economic impact of livestock disease [41, 42].

The framework for quantifying the economic impact of animal disease is defined by Rushton et al. [41, 42] (Figure 2). A targeted literature review was performed to parameterize impact on a per-case basis for the disease–species combinations in the regions of interest.

Table 3 lists the expenditure and loss items included in the disease impact estimates for each of the three diseases. FMD can produce a reduction in milk yields over the lactation period of up to 20% [44], a reduction in sale value of cattle of 37% [45], and the loss of price premiums on exports [46]. Knight-Jones and Rushton [43] aggregated the published literature on FMD direct impacts and response costs in endemic regions and estimated that on average a cost per case of USD $100 is most likely, with a maximum of $370 per case in higher yielding breeds [47]. Foot and mouth disease vaccination strategies vary by country, with most countries in the sample applying a strategic vaccination strategy, including response to outbreak events, with or without routine vaccination against prevalent virus strains [48]. No countries in the sample reported slaughtering animals in response to FMD during the reporting period.

For ASF, total slaughter of at-risk populations was observed within WAHIS data, although reporting from West African countries did suggest variation in slaughter rates. This is contradicted by literature reports; however [31], as a result a 100% slaughter rate is applied in the model for all ASF-exposed populations. No vaccine for ASF has yet been applied in the field. Farms are most often completely depopulated and may be prevented from restocking for an extended period [32]. The increased demand for replacement animals in affected areas can cause price increases in live pig prices of up to 100%, while commercial farms may enhance biosecurity, quarantine, and screening of new stock [31].

HPAI almost universally resulted in culling of all exposed animals in the WAHIS data. In terms of other response costs, HPAI vaccines are available but tend only to be used when stamping-out methods have failed [49]. Countries such as those in this sample would typically achieve coverage of up to 40% of the population. This is therefore applied as a top limit in this analysis. According to McLeod and Hinrichs [50], downtime between depopulation and restocking and resumption of business was parameterized at 3 months for broilers and layers and 9 months for breeder flocks. As a zoonotic disease, HPAI outbreaks can have dramatic effects on demand for poultry meat and eggs as well as pullets [24, 50–52] and this is reflected in the model and given a range to represent what has been observed in case study countries. Price recovery was assumed to be linear and return to preoutbreak levels at 3 months postoutbreak. The individual economic and response parameters are listed in Table 4.

### 2.3. Cost–Benefit Estimation

The production system models for cattle, pigs, and chickens were used to estimate the gross value of production of meat, milk, and eggs, direct losses to production, and additional response costs and revenue foregone under the following three disease conditions:

- no disease event,
- major disease event, and
- limited disease event.

In order to assess the benefits of averting a major disease outbreak event, the economic costs of investment were combined with estimates of disease impact over time in a cost–benefit analysis framework.

Within each of these categories of event, uncertain parameters were placed into the model at minimum and maximum values. This established the total range of possible lost value from each system under each disease scenario. These ranges were then applied in Monte Carlo simulation in the R Statistical Software (10,000 iterations) as pert distributions with a mode, minimum and maximum. From these distributions, the financial benefit of averting a major disease event was estimated. Within the model, the occurrence of an outbreak in a given species in a given year is considered to be an independent event, such that outbreaks can occur in none, single, or multiple species, and at no, single, or multiple times over the 10-year evaluation period. By assessing the cost saved across the range of probability combinations, the benefit of efficient emergency response is calculated as the present value of the sum of total losses averted over the 10-year period. Combined with the two alternative cost scenarios net of the baseline, net present values (NPVs) of investment scenarios were calculated according to Equation (1) at a 5% discount rate. A probability matrix was constructed, with annual probability of a major disease event on one axis, and probability of averting a major event on the other.

NPV of an investment in animal health preparedness is presented in Equation (1):

(1) $$\begin{array}{c} NPV=\sum {PV}_{Benefits}-\sum {PV}_{Costs}. \end{array}$$

This distribution of outcomes was analyzed to determine the break-even frontier across the probability ranges on each access. If *P* (O) is the probability of a major disease outbreak in a given year, and *P* (A) is the probability of averting a major outbreak, then NPV was observed to be linear in both when separated. As such the breakeven point, i.e., where NPV is zero, in terms of the probability of averting a major outbreak and probability of an outbreak occurring can be described as a frontier with two parameters *m* and *c*, for all values of *P* (O) and *P* (A) from 0 to 1:

(2) $$\begin{array}{c} P\left(\text{A}\right)=\frac{-c}{m\cdot P\left(\text{O}\right)}. \end{array}$$

## 3. Results

### 3.1. Outbreak Impact Distribution

Major disease outbreaks as defined by the method described caused in the region of 10% of gross value of output to be lost for HPAI, 7% for FMD, and 25% for ASF. These figures had a large range across the countries concerned, with the intensity of production and relative productivity of the livestock populations being influential over the total proportion of production lost. Mean and spread of all countries by disease are given in Table 5.

### 3.2. Cost–Benefit Analysis of Investing in Emergency Preparation under Uncertainty

**Figure 3:**
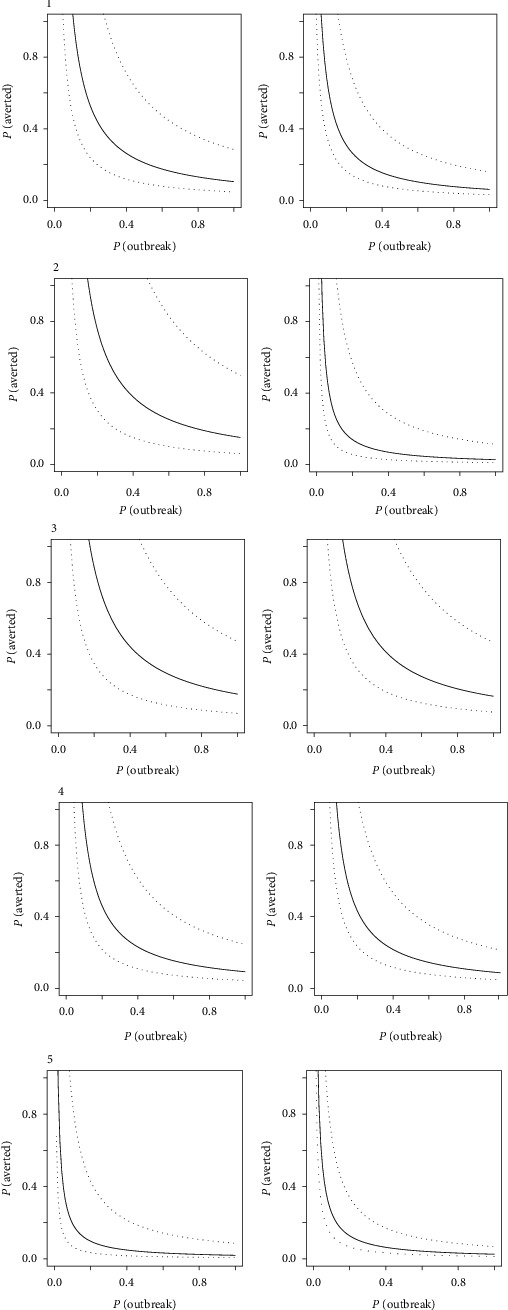
Breakeven frontiers for five countries (1–5) in the East African region based on the probability of a disease outbreak *P* (outbreak) and the probability of averting a major outbreak *P* (averted), comparing a full veterinary service investment (left column) and targeted emergency preparation investment (right column). Median values (solid line) and 95% spread of distribution (dashed lines) are presented. Proximity of the curve to the origin indicates a higher likelihood of breaking even on investment relative to the probability of disease outbreaks and averting a major disease event.

**Figure 4:**
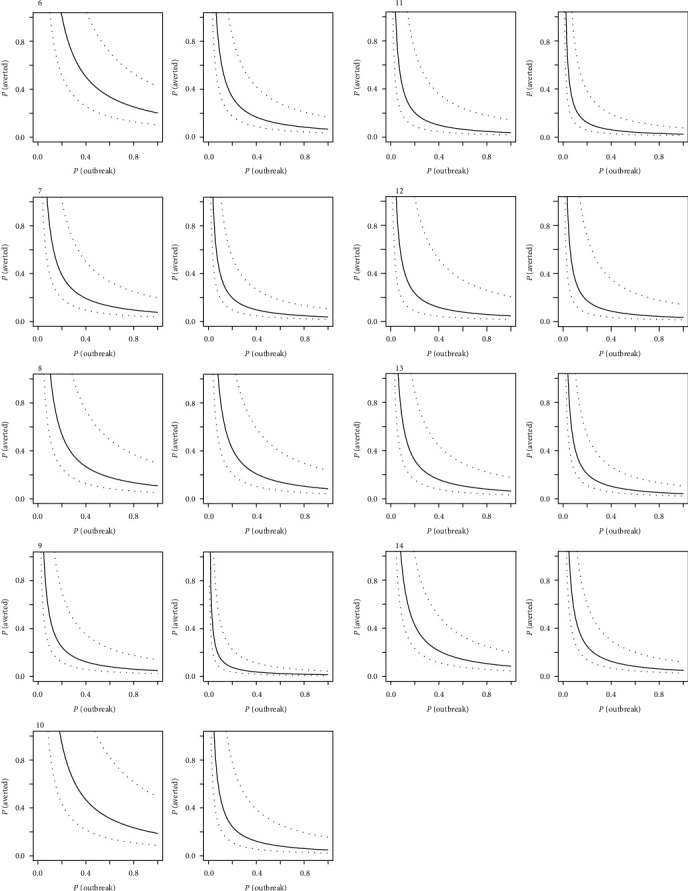
Breakeven frontiers for nine countries (numbered 6–14) in the West African region, with a full veterinary service investment (left) and targeted emergency preparation investment (right) for each country. Median values (solid line) and 95% spread of distribution (dashed lines) are presented. Proximity of the curve to the origin indicates a higher likelihood of breaking even on investment relative to the probability of disease outbreaks and averting a major disease event.

Observing the breakeven frontiers across the three regions (Figures 3 and 4), the area above the frontier curve delimits positive NPV. A curve passing close to the origin therefore represents a situation where investment is more likely to generate positive value. A general trend was observed for more favorable conditions for positive NPVs to be in the South East Asian region, followed by the West African region, and finally the East African region. Due to the sensitivity of budgetary information on VS, and PVS GAP analysis, this break-even analysis provided a means to presenting results without compromising confidentiality.

**Figure 5:**
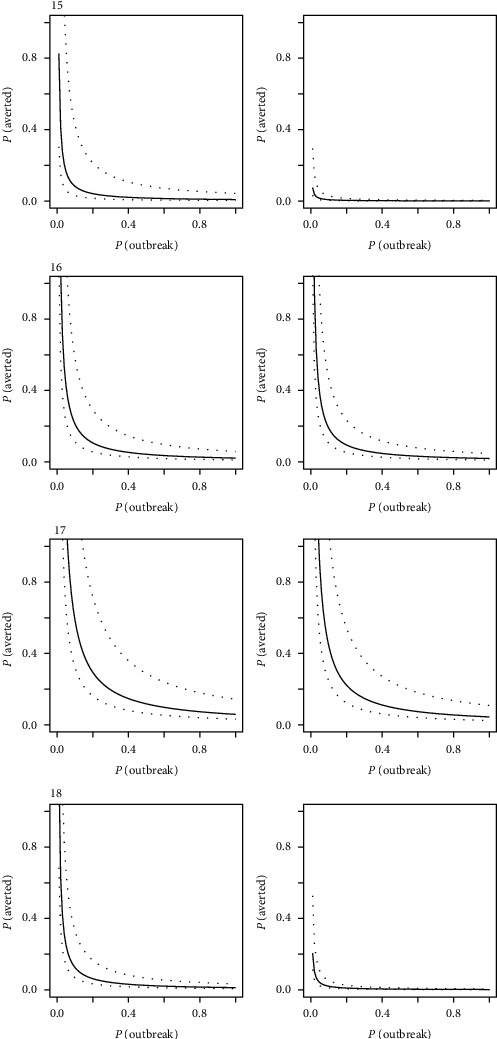
Breakeven frontiers for four countries (numbered 15–18) in the South East Asian region, with a full veterinary service investment (left column) and targeted emergency preparation investment (right column). Median values (solid line) and 95% spread of distribution (dashed lines) are presented. Proximity of the curve to the origin indicates a higher likelihood of breaking even on investment relative to the probability of disease outbreaks and averting a major disease event.

In general, the limited expenditure in the EP-targeted investment produced a more positive economic outcome compared to the wider VS investment. Figures 3–5 illustrate this trend with the distribution of outcomes tending to move closer to the origin in the right-hand column for each country. Individual countries, however, had specific differences that distinguished them from their peers. In Figure 4, for example, countries 1 and 5 would have required high probability of outbreak and high probability of averting a major outbreak, with a relatively narrow confidence band around the median in order to justify a full VS investment simply on emergency management alone. In such a case, a targeted investment would be more economically viable in the context of emergency management, or alternatively, the full VS investment plan would have to be considered for its crosscutting benefits beyond the management of emergencies. These results tended to be influenced most strongly by the size of the livestock population concerned and the level of current investment in VS (i.e., with higher current investment, more targeted investment is needed to enhance EP).

## 4. Discussion

As well as addressing the primary research aims, the results presented facilitate the identification of countries which stand to benefit the most greatly from improvements to their EP. Observing the trends in the output of the cost–benefit analysis, countries with large livestock populations, with increasing levels of intensive production, with a positive trade balance in livestock and livestock products, and with low levels of pre-GAP expenditure on VS stood to benefit the most from improving their EP levels.

Overall, the most significant budget items across the sampled countries' VS were related to laboratory services, contracts with the private sector, passive surveillance systems, and vaccination campaigns. However, considerable between-country variation was noted in post-Gap recommended budgets. In particular, some countries budgeting specific time-limited projects within the Gap-report period which produced disproportionately large budget estimates for the post-Gap scenario. This could result in VS budgets for these countries being 1 and 2 orders of magnitude greater per livestock unit than their peers. These large estimates for the present value of costs reduced NPV outcomes, making additional investment in EP difficult to justify at first glance. However, the benefits of investment to VS are not isolated to emergency management and this has to be borne in mind.

Another issue in setting budgets in LMIC settings is the rate at which budgets are actually executed, representing inefficiency in utilizing resources allocated to particular activities. This is a particularly prevalent issue in health systems in LMICs [53], particularly with reference to capital items such as those needed when developing infrastructure.

A critical aspect to interpreting the results of this study is understanding the likelihood of preventing a minor outbreak developing into a major one. While technical aspects of VS provision, such as access to diagnostic facilities, larger numbers of personnel and access to contingency funding have a clear link to disease management, the emergency management process includes many moving parts for which quantitative analysis is more difficult.

Depending on jurisdiction, the VS may not be the lead agency and will be reliant on other bodies for coordination. This may link to skills outside of typical VS training such as working with law enforcement to enforce disease control measures, supply chains, logistics, and incident command structures. The PVS process acknowledges this in CC 1-6 *coordination capability of the Veterinary Service* (*external*) but given the complexity of assessing such a capability across many different countries it remains poorly understood how to maximize this. As a result, emergency management does not hinge only on a well-resourced VS but also the other government agencies and private organizations involved in emergency response, and the legal and institutional frameworks necessary to establish roles and responsibilities.

With this in mind, the importance of being able to identify risk exposures in livestock systems through risk analysis and respond quickly and effectively to threats in coordination with other agencies will be contingent on scenario planning and simulation exercises for development. A recent investigation, however, has identified that despite many countries having contingency plans for animal disease emergencies, less than half of those have held simulation training exercises in the last 10 years [11].

Disease impact as estimated using the limited framework applied here, however, included only specific direct and additional costs. The wider economic and livelihood impacts of livestock mortality and morbidity, food security and human health consequences were not considered Zoonoses, in particular, were not modeled, despite HPAI itself being of considerable risk to human health with a case fatality rate exceeding 50% [54]. The intricacies of modeling transmission through the food chain was not within the scope of this project and would have weighted outcomes further toward countries with large poultry sectors due to this zoonotic risk.

In terms of benefits, the models also do not capture the knock-on effect of investing in infrastructure and human capital. Much of the PVS Gap budget recommendations are focused on training, communication, and physical resource investment, which can be applied in a crosscutting fashion within the animal health system. As a result, where VS often lack the resources to perform basic functions many countries face significant endemic disease burdens, raising the resources and expertise within the animal health system is likely to result in crosscutting benefits in such a case.

From the perspective of international trade, the trade in live animals, particularly in the East and West African and Middle East regions must be noted. The introduction of a previously unknown pathogen into one of these populations could have serious additional consequences for national incomes and livelihoods, as well as for meeting people's demand for livestock products, but as the majority of countries within the sample were net importers of meat and milk, a quantitative analysis of international trade impacts was not within the scope of this study.

In many of the countries concerned, natural disease outbreaks occur at a relatively high frequency [55], and the possibility that a proportion of these outbreaks are preventable is an important point to consider. While major outbreaks are less frequent, the human and political cost of failing to minimize their occurrence can be enormous. In the case of a deliberate release of pathogen, it is reasonable to assume that a human agent would be more efficient at generating an epidemic than natural agents, increasing the probability of a major rather than a limited outbreak.

The same probability distribution was applied to all countries in this analysis. This does not take into consideration the differences in risk exposure in some countries due to physical or human factors, practices such as transhumance, and physical factors such as seasonality of weather and grazing availability. A more detailed interpretation of these results should take into consideration these local factors as part of any new policy-making process. The distribution of outbreaks reported to WOAH was also accepted as representative. This may not be the case since underreporting of disease could occur. It could also be supposed that underreporting would be biased toward smaller rather than larger outbreaks, meaning large outbreaks occur less frequently within the true distribution of outbreak size. Without understanding the magnitude of underreporting, however, it is difficult to say whether any material effect on the results of the analysis would be produced.

The PVS Gap data are also limited in the range of country reports that are available. Given the remit of the PVS system, the dataset includes more countries with low-capacity VS. At the outset, it was intended to include the Middle East as a region of interest. However, given the relative importance and economic focus on other industries in the region, there are relatively few PVS reports available on which to estimate VS costs.

On the methodological side, purchasing power parity (international) dollars were selected for use in the analysis of PVS GAP data. This decision was taken with careful consideration, and arguments can be made both for and against this choice. First, PVS Gap reports varied in the time of data collection, the location, and the reporting currency and so needed to be converted to a common currency and adjusted for inflation. A common currency allowed comparison between countries. If another currency such as the US dollar were used, the differences in spending power of the dollar in different economies would introduce a systematic bias in the results even after adjustment for inflation and exchange rates over time. Purchasing power parity accounts for this difference by calculating the cost of a basket of goods in each country and benchmarking against the cost of that basket in the United States. The issue in the context of this report is that the purchasing power parities method does not include the provision of government services in the hypothetical goods basket from which it is estimated, and therefore cost estimates are subject to a level of uncertainty as to their accuracy.

Further investigation of figures for trade in livestock species and product categories might shed further light on the drivers for investment in investment in VS. Within the study sample, those countries with positive trade balance in livestock and products appeared to be in less need of capital investment and have larger annual budgets in relative terms at the time the GAP Analysis was performed. This may reflect efforts on the part of governments to protect the capital value of livestock populations as they are an important generator of economic activity and revenue.

Additional modeling of VS funding could be made by integrating analysis with WAHIS outcomes. The WAHIS database tracks disease outbreaks and country status over time. Looking for associations between particular disease statuses or longitudinally looking at particular events for influence on VS budgets could be a useful tool to understanding the drivers of funding for VS.

## 5. Conclusion

The results presented showed many countries stand to benefit from relatively small improvements in their emergency preparation status and provided this translates into a reduced probability of a major outbreak occurring. Given that many countries in the sample were endemic for many significant animal pathogens, and emergency preparation is in many respects crosscutting, the model does not fully capture the potential to derive economic benefits from improvements to VS competency. In some countries, the returns on investment appear relatively small, yet the impact of managing disease across larger geographical regions was not included. There are strong arguments that the next step of this work needs to include the wider global benefits of managing animal diseases—the global public good and also the national benefits of having a strong animal health system that impacts positively on the management of the endemic diseases in the livestock. These two elements would provide a much stronger impression on the returns on investment of the VS.

## Figures and Tables

**Table 1:** Descriptive characteristics of livestock populations and economy in sample countries undergoing PVS Gap analysis in the period 2007–2013, grouped to preserve anonymity.

ID	Livestock units per capita (2017)	GDP per capita at time of analysis	Estimated proportion of chickens in commercial systems (%)	Estimated proportion of pigs in commercial systems (%)	Net exporter (by total value of trade)	Estimated Pre-Gap Veterinary Services expenditure per livestock unit
1	Less than 0.2	Less than $500	5	5	No	More than $1
2	More than 0.5	Less than $500	13	7	Yes	Between $0.1 and $0.5
3	More than 0.5	$500–$1,000	26	12	Yes	More than $1
4	Between 0.2 and 0.5	$500–$1,000	15	8	No	More than $1
5	Between 0.2 and 0.5	$500-–$1,000	14	8	Yes	Between $0.5 and $1
6	Between 0.2 and 0.5	$500–$1,000	17	9	No	More than $1
7	More than 0.5	$500–$1,000	13	7	No	Less than $0.1
8	Less than 0.2	$1,000 or more	32	13	No	More than $1
9	Less than 0.2	$1,000 or more	36	15	No	Between $0.1 and $0.5
10	Between 0.2 and 0.5	$500–$1,000	17	9	No	Less than $0.1
11	More than 0.5	$500–$1,000	16	9	Yes	Between $0.5 and $1
12	More than 0.5	Less than $500	5	5	Yes	Between $0.1 and $0.5
13	Between 0.2 and 0.5	$1,000 or more	29	12	No	More than $1
14	Less than 0.2	$500–$1,000	11	7	No	Between $0.5 and $1
15	Less than 0.2	$1,000 or more	95	38	No	More than $1
16	Between 0.2 and 0.5	$500–$1,000	32	14	No	Between $0.1 and $0.5
17	More than 0.5	$1,000 or more	60	22	No	Between $0.1 and $0.5
18	Between 0.2 and 0.5	$500–$1,000	52	20	No	Less than $0.1

**Table 2:** Epidemic parameters for three diseases of livestock extracted from global reports to WOAH–WAHIS over the years 2015–2019.

Variable	Region	Species	Outbreak size	Value	Source
Proportion of national population at risk	Global	Pigs	Major	0.0943	99% of empirical distribution
			Limited	0.000043	50% of empirical distribution
		Chickens	Major	0.0659	99% of empirical distribution
			Limited	0.00017	50% of empirical distribution
		Cattle	Major	0.131	99% of empirical distribution
			Limited	0.00045	50% of empirical distribution

Case rate within population at risk	Global	Pigs	Major	1	99% of empirical distribution
			Limited	0.174	50% of empirical distribution
		Chickens	Major	1	99% of empirical distribution
			Limited	0.166	50% of empirical distribution
		Cattle	Major	0.564	99% of empirical distribution
			Limited	0.0552	50% of empirical distribution

Mortality rate within cases	Global	Pigs	N/A	0.976	50% of empirical distribution
		Chickens		0.984	50% of empirical distribution
		Cattle		0	50% of empirical distribution

Slaughter rate of extant population at risk	Global	Pigs	N/A	1	50% of empirical distribution
	Global	Chickens		1	50% of empirical distribution
	Global	Cattle		0	50% of empirical distribution

Major and limited outbreaks are defined by the 99th and 50th percentiles of the empirical distribution for each parameter.

**Table 3:** Cost items included in disease impact estimation for each species production system.

Cattle	Pigs	Chickens
Direct losses to production	Replacement stock	Replacement stock
Additional response costs including vaccination	Additional biosecurity costs	Carcase disposal costs
See Knight-Jones and Rushton [43]	Carcase disposal costs	Vaccination costs
	Revenue foregone in downtime	Lost revenue from reduced sale price
		Lost revenue from downtime

**Table 4:** Economic and production parameters applied within the disease costing models for each species.

Variable	Value
Chickens	

Proportion of chicken population in intensive production	Approximated to Gilbert et al. [22]
Vaccination coverage HPAI	0–40 (major) 0 (limited)
Time to price recovery	3 months (major) 0 months (limited)
Affected population downtime	Breeders 9 months Layers and broilers 3 months
Price change, eggs	−50% (−25% to −75%)
Price change, broilers	−60% (−40% to −80%)
Price change, pullets	−75% (−60% to −90%)
Egg price ($/kg)	2
Broiler price (mature) ($/head)	9
Layer pullet price ($/head)	1.5
Breeder price ($/head)	10
Point of lay hen price ($/head)	6.5
Broiler price (all ages) ($/head)	5.25

Cattle	

Direct and response costs per case ($/head)	100 (100–370)
Meat price ($/kg)	4 (3–5)
Milk price ($/kg)	0.4 (0.3–0.5)

Pigs	

Proportion of chicken population in intensive production	Approximated to Gilbert et al. [22]
Pork price ($/kg)	2.5 (1.65–3.35)
Replacement breeder price ($/head)	400 (268–532)
Replacement grower price ($/head)	Half of sale price finished pig (Estimated from average slaughter weight (FAOSTAT))
Time to restock	1 year
Biosecurity cost ($/head)	25
Carcase disposal ($/head)	1.5

**Table 5:** Median, lower, and upper (2.5th and 97.5th percentiles) values of the distribution of change in gross value of production in an outbreak year for each of the diseases and outbreak sizes used to quantify disease impact (mean of all countries).

	Major outbreak			Limited outbreak		
	Lower (%)	Central (%)	Upper (%)	Lower (%)	Central (%)	Upper (%)
HPAI—chicken	−7.12	−9.42	−12.82	−0.0078	−0.008	−0.010
FMD—cattle	−5.46	−6.83	−33.7	−0.0018	−0.0023	−0.011
ASF—pigs	−19.67	−24.67	−32.52	−0.009	−0.011	−0.015

## Data Availability

FAO, United Nations, and World Bank data were used to support this study and are available at https://www.fao.org/faostat/en/#data, https://comtradeplus.un.org/ and https://databank.worldbank.org. These datasets are cited at relevant places within the text as references. Selected data from the Performance of Veterinary Service Review is published with permission of the host country here at https://www.woah.org/en/what-we-offer/improving-veterinary-services/pvs-pathway/#ui-id-5. Some data from the WOAH PVS and PVS GAP program are not publicly available and are held by the World Organisation for Animal Health (WOAH), Paris. Further information is available from the corresponding author.

## Supplementary Materials

Supplementary Materials A table of relationships assumed between critical competencies in different versions of PVS and PVS Gap Analysis over time is used to allow the aggregation of expenditure data from different periods. Table S1: critical competency inclusion, priority level for emergency preparation (EP) and numbering across various iterations of Gap analysis procedure.
