# Supplementary material for: A Cost–Benefit Analysis of Preparing National Veterinary Services for Transboundary Animal Disease Emergencies
Source: Transbound Emerg Dis. 2023 Nov 4;2023:1765243. doi: 10.1155/2023/1765243 (PMC12017056; doi:10.1155/2023/1765243)
Supplement: Supplementary Materials — A table of relationships assumed between critical competencies in different versions of PVS and PVS Gap Analysis over time is used to allow the aggregation of expenditure data from different periods. Table S1: critical competency inclusion, priority level for emergency preparation (EP) and numbering across various iterations of Gap analysis procedure. [file 1765243.f1.pdf]

Supplementary material.

Related critical competencies in different versions of PVS and PVS Gap analysis over time used to aggregate data from different periods.

Table S. 1. Critical competency inclusion, priority level for emergency preparation (EP) and numbering across various iterations of Gap analysis procedure.

Title	EP priority level	FAO GEMP (2021)	PVS Gap (Pre-2010)	Later PVS Gap (2013)
Continuing education	3	I.3	I.3	I.3
Technical independence	4	I.4	I.4	I.4
Internal Coordination	4	I.6.A	I.6	I.6.A
External Coordination	4	I.6.B	I.6	I.6.B
Emergency funding	4	I.9	I.9	I.9
Access to laboratory diagnosis	4	II.1.A	II.1	II.1
Suitability of the laboratory system	4	II.1.B	-	-
Laboratory quality assurance		-	II.2	II.2
Risk analysis	4	II.2	II.3	II.3
Passive epidemiological surveillance	4	II.5.A	II.5.A	II.5.A
Active epidemiological surveillance	4	II.5.B	II.5.B	II.5B
Emergency response	4	II.6	II.6	II.6
Regulation, authorisation and inspection of premises	3	II.7.A	II.8	II.8.A
Veterinary medicines and biologicals	4	II.8	II.9	II.9
Identification and traceability of animals	3	II.12.A.	IV.6	II.13.A
Identification and traceability of products	3	II.12.B	IV.6	II.13.B
Communication	4	III.1	III.1	III.1
Consultation with stakeholders	4	III.2	III.2	III.2
Accreditation, authorisation and delegation	3	III.4	III.4	III.4
Participation in joint programmes	4	III.6	III.6	III.6
Legislation – coverage	4	IV.1	IV.1	IV.1
Legislation – enforcement and compliance	3	IV.2	IV.2	IV.2
